# Significance of immunohistochemistry biomarkers in prediction of malignant transformation of oral lichen planus: A systematic review

**DOI:** 10.4317/medoral.25491

**Published:** 2022-08-17

**Authors:** Aisha A H Al-Jamaei, R V Subramanyam, Marco N Helder, Tymour Forouzanfar, Mohammad Ruslin, Erik Van der Meij, Jan G A M de Visscher

**Affiliations:** 1 Department of Oral and Maxillofacial Surgery/Oral Pathology, AmsterdamUMC-location VUmc/Academic Centre for Dentistry Amsterdam (ACTA), Amsterdam, The Netherlands; 2 Department of Oral Surgery and oral Medicine, College of Dentistry, Al-Razi University, Sana’a, Yemen; 3 Department of Oral Surgery and Oral Medicine, College of Dentistry, Ibn Al-Nafis University, Sana’a, Yemen; 4 Department of Oral Pathology, K M Shah Dental College and Hospital, Sumandeep Vidyapeeth, Vadodara, Gujarat, India; 5 Department of Oral and Maxillofacial Surgery, Faculty of Dentistry, Hasanuddin University, Makassar, Indonesia; 6 Department of Oral and Maxillofacial Surgery, Medical Centre Leeuwarden, Leeuwarden, The Netherlands

## Abstract

**Background:**

Oral lichen planus (OLP) is a chronic inflammatory disorder with increased risk for malignant transformation. Biomarker validation is a pivotal step in moving newly discovered biomarkers towards clinical implementation. We performed a systematic review of studies on biomarkers related to OLP, wherein biomarkers have been described in at least two independent studies. Our aim was to determine whether any of these biomarkers might be promising in predicting the increased risk of malignant transformation of OLP.

**Material and Methods:**

We searched the following databases until August 2021: PUBMED, EMBASE, and Web of Science. Due to high heterogeneity, a qualitative rather than quantitative assessment was conducted. Only proteins that consistently showed a significantly high level of expression in neoplastic tissues versus OLP in two or more publications were considered as promising markers.

**Results:**

Initial database researches identified 1671, of which 24 articles were included in the final analysis. The most frequently reported proteins were p53, Bcl-2 and Ki-67, though there were controversies. PCNA and P21 were the only proteins that showed consistent evidence of clinical usefulness as cancer predictors to be considered as promising markers. Extensive methodological variations in the evaluation of expressions and statistical analyses of the included markers were observed, which hampered comparisons of the results.

**Conclusions:**

Multiple levels of heterogeneity with a scarcity of high-quality studies were identified. PCNA and P21 were identified as promising predictive markers for evaluating the risk of malignant transformation of OLP, but they require further validation. The focus of future research on validation of predictive biomarkers of OLP should be considered as a high priority because it will accelerate the introduction of newly discovered markers into the clinical setting.

** Key words:**Oral lichen planus, Immunohistochemistry, biomarkers, malignant transformation, PCNA, P21, p53, BCl-2.

## Introduction

Abbreviations: Oral lichen planus (OLP), Immunohistochemistry (IHC), Malignant transformation (MT), Oral epithelial dysplasia (OED), Oral squamous cell carcinoma (OSCC), Malignant transformation rate (MTR).

Oral Lichen planus (OLP) is a relatively common disease of the oral mucosa and appears to be an immunologically-mediated chronic inflammatory reaction to an unknown antigen (1). A recent review of the global prevalence of OLP showed a pooled prevalence of 1.01% with a marked variation in regional distribution (2). OLP has a prolonged clinical course, extending for years and sometimes even lifelong and typically affects middle-aged adults, with a Female: Male ratio of 2:1 (3). Clinically six types of OLP have been identified: reticular, atrophic/erosive, plaque-like, papular, ulcerative, and vesiculo-bullous. The coexistence of more than one subtype of the disease is also common (3). Although, there are various local and systemic treatment modalities reported for treating painful OLP, the available evidence suggests that topical corticosteroids are most effective (4).

The overall malignant transformation rate (MTR) of OLP varies between 0.5 -1.2 % (5) but has been reported to be as high as 2.8% (6). The MTR varies among different clinical types but generally higher in atrophic and/or erosive types. Currently, the absence or presence of oral epithelial dysplasia (OED) is still the mainstay for malignant transformation (MT) risk assessment (7). However, histopathological observations do not provide a clear distinction between dysplasia developed in OLP and lichenoid reaction developed in oral dysplasia and are also subject to inter and intra-observer variability (8). Additionally, presence of epithelial dysplasia and its grade is not a reliable indicator of the biological behavior of these lesions. A recent meta-analysis showed that 6% of dysplastic OLP progressed to carcinoma, but also that less than 1.5 % of non-dysplastic lesions progressed to oral cancer (7). Since this is prognostically important, there is an urgent need for novel biomarkers that can accurately predict the malignant potential of OLP lesions.

One relatively simple and affordable technique to study biopsies of OLP and detect pathogenic molecular abnormalities in gene expressions at the protein level is Immunohistochemistry (IHC). IHC is the most common technique that is used to visualize the intracellular as well as the cell membrane proteins, thereby providing a real reflection of what is going on at the cellular level (9).

There is, indeed a three-level evidence hierarchy for biomarker validation, ranging from exploratory to validated to clinically useful. To qualify as a useful biomarker, it is essential to successfully pass them all. A exploratory biomarker is defined as any biomolecule identified in one discovery publication, whereas validation is based primarily on confirming a discovery biomarker’s finding in at least two independent studies (10). It is a second and critical step in moving any biomarker towards clinical implementation. To our knowledge, this review is the first to focus on proteins that have been validated in at least two independent studies. We evaluated studies with potential biomarkers to predict risk of malignant transformation in OLP, and in which these markers demonstrated a consistent and significant difference in their expression between OLP and OSCC or OED. Our aim is to help researchers in identifying and pursuing the promising biomarkers for further evaluation and validation studies.

## Material and Methods

This systematic review was conducted and reported following PRISMA-protocol guidelines (11). However, it could not be registered at the International Prospective Register of Systematic Reviews (PROSPERO) (12), because the entire data was completely extracted and analyzed before registration, which is against PROSPERO requirements.

- Search strategy

Potentially eligible studies were identified in a search of only indexed databases like PubMed, Embase and Web of science. The following keywords were used: “oral lichen planus,” “OLP,” “oral squamous cell carcinoma,” “OSCC,” “oral cancer,” “oral carcinoma,” “epithelial dysplasia,” “oral normal mucosa,” “oral hyperkeratosis,” “malignant transformation” and all possible combinations. The search results were supplemented with manual searching of reference lists to identify publications not captured by computerized searches. There was no restriction regarding year of publication. The last search was conducted in August 2021.

- Studies selection

Abstracts that focused on the malignant potential of oral lichen planus compared to that of the normal oral mucosa, OSCC, and OED, by their ability to express specific proteins, were identified by the first author. Subsequently, full-text articles of the selected abstracts were retrieved and assessed independently and blindly by the first and second authors (AAA and RVS) based on specific eligibility criteria. The list of the selected studies prepared by both authors then compared and only those achieved a good agreement were included.

- Inclusion Criteria

1) Studies including patients with a confirmed histopathological diagnosis of OLP

2) Studies reporting protein expression with a recognized classification/grading system

3) Studies clearly comparing protein expression between OLP and neoplastic or dysplastic tissues

4) Studies focusing specifically on OLP and its malignant

- Exclusion criteria

1) Studies that included patients with clinic signs of oral lichenoid lesions

2) Studies that also focused on oral potentially malignant lesions other than OLP, such as

 leukoplakia, erythroplakia, or oral submucous fibrosis

3) Studies with a sample size ≤ 10 cases

4) Studies published in languages other than English

5) Studies using animal models or tissues, or various biological samples rather than human tissue

6) Case reports, systematic reviews, unpublished studies, and duplicate publications

- Data acquisition

The following data for each study were extracted from full-text articles by the first and second authors (AAA and RVS): study molecules, biomarkers’ functions, sample size, expression rate, odds ratio if reported clearly, diagnostic criteria, and significance of expression in OLP vs normal mucosa and versus OED or SCC. With respect to the biomarkers that were reported in two or more publications, our focus was to assess the consistency and significance in their clinical relevance. In other words, when two or more studies showed significant differences in the expression of specific biomarkers in OED or SCC compared to OLP, they would be considered as promising markers in predicting the risk of malignancy.

- Quality assessment

The quality of the selected biomarkers studies was independently assessed by the first two authors on the basis of the criteria as formulated in the Reporting Recommendations for Tumor Marker Prognostic Studies (REMARK), which comprise 20 items (13). Each item of this guideline encompasses several aspects. One point was given to the item when all of its aspects were clearly stated, 0.5 point was attributed if some but not all aspects were mentioned, and 0 point were given when the item was not reported. Based on the total scores, the studies were subdivided into three groups: studies with a score of 15-20 were assigned as high reporting quality, studies had a score of 5 -14.5 were considered to have an average reporting quality, and low reporting quality when the score ≤ 5. Disagreements were resolved by discussion.

## Results

**Figure 1:**
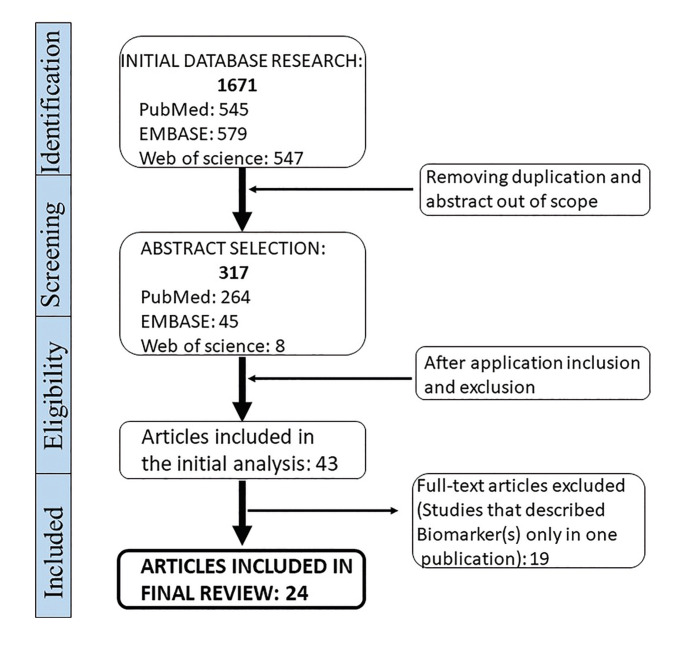
Diagram of studies selection.

As shown in Fig. 1, the search strategy yielded 1671 potentially relevant studies, from which 1626 were excluded as they did not meet the eligibility criteria. A total of 43 papers, strictly met the inclusion criteria. However, because validation is the first step in moving newly discovered biomarkers towards clinical implementation, we focused on biomarkers that have been validated in two or more publications. Therefore, out of 43 eligible studies, only 24 were included in this review for data extraction. The studies described a total of 1,802 participants, and the study sample size ranged from 37 to 164 patients. Of the participants, 46% were males and 54% were females. The studies were conducted in different geographic areas, of which 11 studies were situated in Asia, 7 in Europe, 5 in America, and one study in Oceania.

The REMARK reporting assessment is summarized in Supplement 1. All included studies had an average REMARK score which ranged between 9 and 13 points. The statistical methods varied strongly among the included studies, and only one study clearly estimated the risk of MT and provided the odds ratio (14). In all studies, no information was provided regarding the treatment that patients received.

Table 1 presents an overview of all the reported markers and the differences in the rates of their expression between healthy mucosa, OLP and OED or OSCC.

**Table 1:**
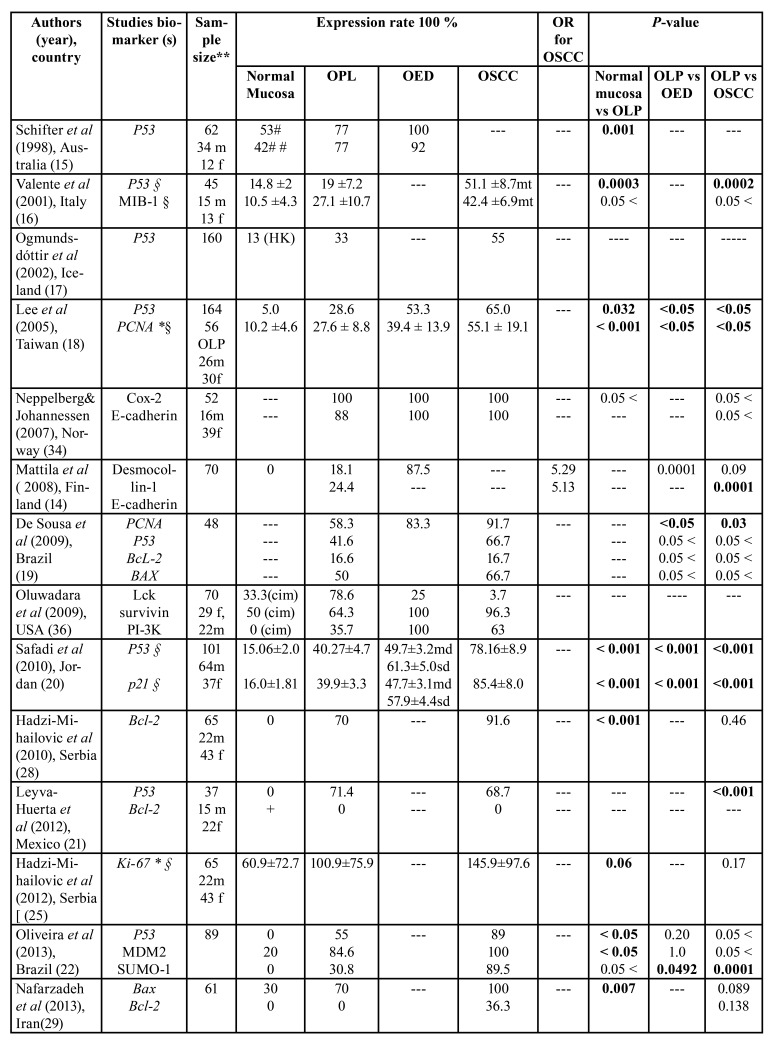
Characteristic and summary data of the included studies (n=24).

**Table 1 cont.**
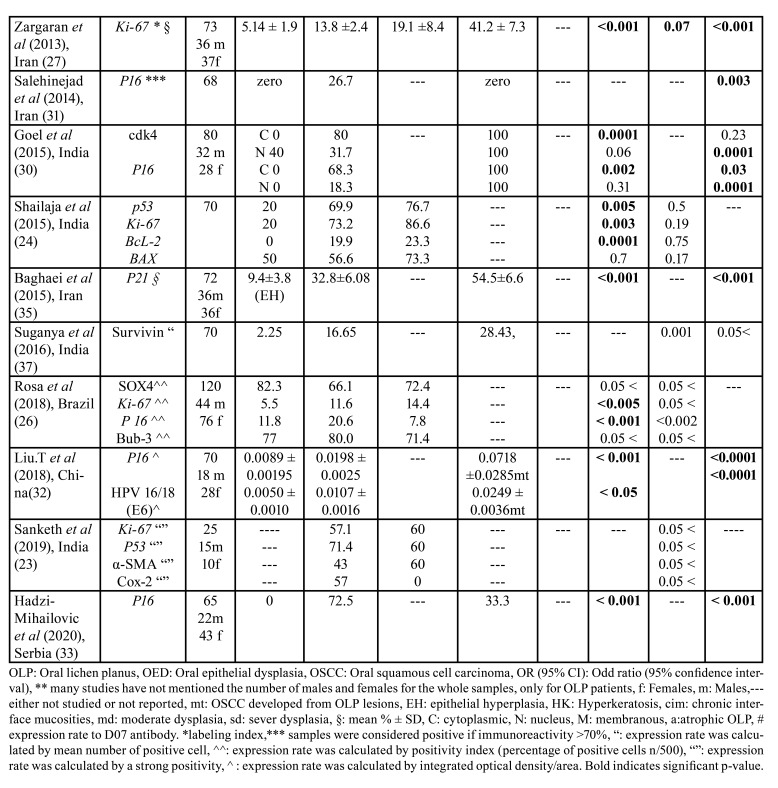
Characteristic and summary data of the included studies (n=24).

**Figure 2:**
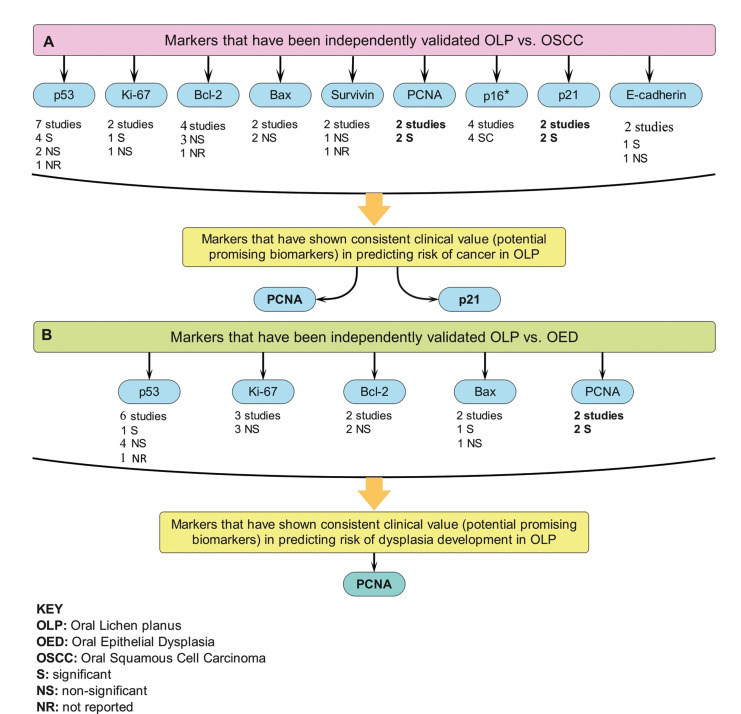
A diagram illustrating the promising IHC markers in predicting risk of cancer (a) or risk of dysplasia development (b) in OLP.

Sixteen of these papers assessed the difference in expression of specific proteins including samples of OLP and OSCC, four studies examined expression in OLP, OED, and OSCC, while four studies focused on difference of expression between OLP and OED. A total of 22 biomarkers was evaluated, and the most frequently studied protein was p53 which was reported in 10 studies (15-24), followed by Ki-67 (23-27), and Bcl-2 (19,21,24,28,29) which were each assessed in 5 studies. With regard to the other markers, p16 was reported in 4 studies (30-33), while E-cadherin (14,34), p21 (20,35), PCNA (18,19), and Survivin (36,37) were reported in two studies each. Out of seven studies that specifically evaluated the association between p53 expression and the risk of SCC, four studies showed a significant association (16,18,20,21), two showed no relation (19,22), while the last one did not report statistical analyses (17) (Fig. 2). Additionally, four out of five studies showed no significant association between the degree of p53 expression and the risk of the presence of epithelial dysplasia (19,22-24). Overall, these results revealed that the positive immunostaining value of p53 protein in predicting the risk of OSCC is inconsistent and not likely to be useful as a predictive marker in MT.

Although virtually all of the included studies presented merely univariate analyses, there was one study that conducted multivariate regression analyses with measurement of odds ratio or hazard ratio (14). The study revealed that the risk of MT in patients with E-cadherin positive expression was significantly higher than those with E-cadherin negative expression (OR = 5.13 95% CI [3.3- 8.1]). Regarding dysplasia, only desmocollin expression showed the highest reported risk of neoplastic progression (HR = 44.13; 95% CI 3.7-525.6) (14).

Of the identified proteins, only PCNA (18), (19) and p21 (20), (35) revealed consistent and significant high level of expression in dysplastic and neoplastic tissues compared to OLP in two or more independent studies (Fig. 2). Thus, based on the current study criteria, these two markers could probably be useful in identification of the risk of MT, though all reported observations were based on univariate, and not multivariate, analysis.

## Discussion

Currently, the absence or presence of epithelial dysplasia is the gold standard for assessment of the risk of MT in OLP (7). However, this standard is still a challenging because it lacks the intra-/inter-observer reproducibility even with a new binary system of grading OED (38) and development of cancer in OLP lesions without epithelial dysplasia has also been reported. Accordingly, there is an urgent need for identification of reliable biomarkers to detect the risk of cancer development in OLP to allow closer and frequent monitoring for the patients, improve early cancer detection and thus increase survival rates. In this review, we performed an extensive search for IHC markers that consistently showed significant high level of expression in neoplastic tissues compared to OLP in two or more publications. Only PCNA and p21 were found as individual markers that might aid in prediction of MT of OLP, but this warrants further validation for their clinical values. We also found that the immunostaining expression of p53 in predicting risk of cancer in OLP patients remains controversial, while it is more likely to be ineffective in predicting lesion’s progression to dysplasia. Furthermore, REMARK report score shows substantial and multilevel of heterogeneity across the included studies with overall paucity of good-quality studies.

It is well-documented that loss of proper control on cell cycle is the main engine in driving cells to neoplastic formation(39). Thus, it was not surprising that the two markers (PCNA and P21) identified in this review were related to cell cycle regulation. While PCNA well-correlates with cell proliferation (40),p21 acts as tumor suppressor protein (39). However, there is evidence that p21 might also act as an oncogene under certain conditions (41).

Previous studies have suggested that PCNA expression plays a crucial role in oral carcinogenesis as it is sequentially increased in the following order: normal oral mucosa, oral hyperkeratosis, OED and OSCC (42,43). In accordance with this observation, we found two IHC studies that demonstrated a significant higher expression of PCNA in OED and OSCC compared to OLP and normal oral mucosa (18,19). Of interest, Lee *et al*, observed a significantly higher level of PCNA expression in atrophic OLP lesions compared to hypertrophic lesions. This, in turn, correlates with the clinical observations that higher rates of MT were found in patients with atrophic OLP lesions (44). Overexpression of PCNA has also been reported to be associated with neoplastic progression in oral submucosa fibrosis, which is classified by WHO as an oral potentially malignant disorder (6). The observed correlation between increased PCNA expression and MT may be due to the fact that the higher the fraction of growth, the higher the chance to acquire mutations and to eventually develop malignant phenotypes. Collectively, as proliferation is one of the hallmarks accompanying cancer development, it can therefore be assumed that increased PCNA proteins expression in OLP lesions is an ominous step that transforms a premalignant lesion to a malignant lesion. Because this result is only based on two publications, this might be insufficient to prove the prognostic role of this protein. Further validation is definitely needed.

p21 is a downstream effector of p53 that acts as a potent tumor suppressor through inhibition of cyclin-dependent kinase activities (45). In this context, lack or downregulation of p21 expression would be qualified as a logical event accompanying cancer progression. However, this is not the case with oral cancers (46). In a study positive staining of p21 was found to increase as OED progress to OSCC (47). In the current survey, we found two studies that reported a significant and gradual increase in p21 expression from normal mucosa to OED to OSCC (20,35). In terms of staining intensity, both studies revealed moderate and strong staining of p21 in OSCC compared to weak and negative staining in OLP. In light of these observations, it seems that activation of p21 in oral carcinogenesis might occur by a p53-independent pathway. A recent review has suggested that cell cycle regulators have the ability to perform different actions in different subcellular locations (48). Thus, it is possible during tumor progression that p21 is translocated to the cytoplasm and acquires an oncogenic property, substantiating its high expression in OED and OSCC. Overall, there seems to be some evidence supporting the usefulness of p21 protein in the prediction of MT in patients with OLP, though further validation with due attention to p21 localization in the tissues is needed.

Our study revealed that the effectiveness of p53 as a prognostic marker of cancer risk development remains controversial. This observation is consistent with a study that showed that p53 protein expression is questionable in predicting MT of oral leukoplakia (49). Nonetheless, a long-term follow-up study of OLP patients revealed enhancement of p53 expression in the lesions that progressed to OSCC, suggesting involvement of this protein in MT (16). Here, it is worth mentioning that the detecTable level of p53 protein in IHC often represents the mutant p53 gene, which is more sTable than the wild-type, though existence of such an association is not always necessary (50). This might explain the suggestion that the presence of p53 gene mutations, and not of wild type p53 protein, is an indicator for potential malignancy (51). One reason for conflicting results about p53 protein expression is differences in clinical characteristics of patients, particularly differences in cancer causing agents. It is well-known that p53 expression increases substantially with tobacco in head and neck cancer (52,53). Therefore, our finding supports the idea of validation for p53, bearing in mind to standardize patients’ clinical features.

In this review different sources of heterogeneity were found where an important one being the clinical characteristics of the samples. The vast majority of the studies (22 out of 24 studies) compared proteomic of OLP samples to de novo OSCC samples that were not the result of MT of OLP, while two longitudinal studies compared proteins profiles of OLP that during follow-up eventually developed OED and an OSCC (16,32). This is important because the prognostic value of the biomarkers may vary based on the samples’ features. For example, it was found that a downregulation of C-erbB-2 in OED developed in OLP patients, while it increased in OED developed in other lesions (54). Another source of heterogeneity was observed in statistical methodology. As mentioned above, only one study used multivariable prediction model analysis, while the rest conducted exclusively univariate analysis. Indeed, since the most reliable way to determine the increment of the marker in prognosis is by adjusting all other well-known prognostic factors, the confounders might result in huge misinterpretation of the prognostic value of biomarkers.

Our findings should be understood in the context of some limitations. Firstly, we defined the biomarkers to be promising when their predictive value was independently validated in two or more studies. This might result in missing a promising marker that is not yet confirmed in an independent study. One example of such markers that showed very encouraging odds ratio in expecting risk of MT of OLP, but not reproduced so far, are cancer stem cell markers (55). Secondly, we acknowledge that there was an extensive heterogeneity in several levels across the included studies, either in methodology of biomarker detection and assessment, clinical features of the samples, and statistical analyses, which together hinder performing meta-analysis and reaching a definitive conclusion about benefit of using these markers in clinical setting. Another limitation is that our validation approach to identification of predictive biomarkers did not restrict to evaluate protein expression in studies with follow-up, which might have an impact on the robustness of our findings. The reasons for that indeed were the scarcity of such kind of studies (16,32), and none of those studies have validated any biomarker. Finally, our conclusion for these promising cell-cycle regulator markers was based on two publications which might be insufficient. Therefore, further validation for each of these markers or their combination is required. Despite these drawbacks, we believe that our observations will stimulate additional research and draw great attention to the importance of validation of potential novel biomarkers for OLP.

In conclusion, PCNA and p21 have been identified as potentially promising markers for predicting the risk of MT in OLP, but they are in need for further validation in well-designed studies. These studies should investigate the combination of PCNA and p21 along with other encouraging ones markers like cancer stem cell markers or p53 and thus may identify people with OLP at a high risk of MT. Furthermore, standardization of assessment of biomarkers and statistical analyses with REMARK or other prognostic tools in reporting data would be of great value, helping to quantify biomarkers values in predicting the risk of MT. Lastly, the focus on validation of predictive biomarkers of OLP should be considered as a high priority because it will accelerate the introduction of newly discovered markers into the clinical setting.
